# Supplementary material for: Belief updating in decision-variable space: More fine-grained choices attract future ones more strongly
Source: iScience. 2025 Jun 7;28(7):112844. doi: 10.1016/j.isci.2025.112844 (PMC12221758; doi:10.1016/j.isci.2025.112844)
Supplement: Document S1. Figure S1 [file mmc1.pdf]

iScience, Volume *28*

Supplemental information

Belief updating in decision-variable space: More fine-grained choices attract future ones more strongly

Heeseung Lee, Jaeseob Lim, and Sang-Hun Lee

SUPPLEMENTAL INFORMATION

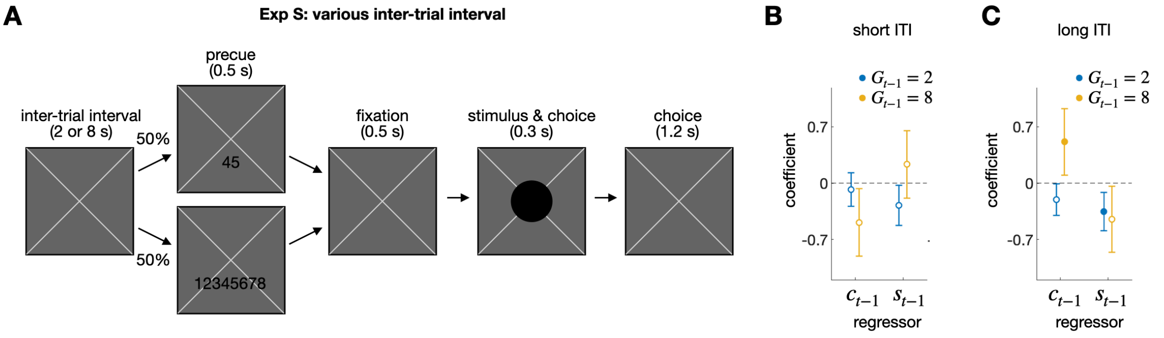

Figure S1. Supplementary experiment (Exp S) with varying durations of ITI, related to STAR Methods

(A) Trial structure. The inter-trial interval (ITI) was randomly assigned either a short (2 s) or long (8 s) duration for each trial. Participants classified the size of a disk into various categories. Data were collected while scanning participants' brain activity inside a 7T MRI scanner. Unlike in the main experiments (Exp 1-7), the threshold for size contrast was not calibrated individually; instead, one fixed distribution was employed for all participants. This led to a size distribution that was significantly wider than the one used in the main experiments, occasionally showing extremely small or large sizes. To align the task difficulty of Exp S with that of the main experiments, we excluded stimuli from the analysis that significantly deviated from the mean, corresponding to the extreme 28 % of the original distribution.

(B,C) Regression of the current choice onto the previous choice (c_{t-1}) and stimulus (s_{t-1}) for the short (B) and long (C) ITI conditions. The regression coefficients are shown separately for decision granularities of 2 (blue circles) and 8 (yellow circles). The filled circles indicate that the corresponding coefficients significantly deviated from 0 after being controlled for FDR.
